# Brucellosis in Colombia: Current Status and Challenges in the Control of an Endemic Disease

**DOI:** 10.3389/fvets.2019.00321

**Published:** 2019-09-24

**Authors:** Lisa M. Avila-Granados, Daniel G. Garcia-Gonzalez, Jorge L. Zambrano-Varon, Angela M. Arenas-Gamboa

**Affiliations:** ^1^Department of Veterinary Pathobiology, College of Veterinary Medicine & Biomedical Sciences, Texas A&M University, College Station, TX, United States; ^2^Departamento de Salud Animal, Facultad de Medicina Veterinaria y de Zootecnia, Universidad Nacional de Colombia, Bogota, Colombia

**Keywords:** brucellosis, *Brucella*, Colombia, livestock, zoonoses, public health

## Abstract

Brucellosis is a zoonosis of nearly worldwide distribution. The disease is considered to be endemic in most of the developing countries with a substantial impact on both human and animal health as well as on the economy. The aim of this scoping review is to provide an overview of the brucellosis status in Colombia and the factors associated with its persistence, to highlight the strengths and gaps of the adopted countermeasures and to supply evidence to policy-makers on the best approaches to mitigate the disease burden. Due to the presence of brucellosis in several susceptible production livestock systems scattered throughout the country, a plan for its control, prevention and eradication was established almost 20 years ago. However, despite extensive efforts, brucellosis prevalence has fluctuated over the years without any trend of decreasing. The restricted budget allocated for brucellosis control is a limiting factor for the success of the program. For instance, the absence of indemnities for farmers results in infected animals remaining on farms which potentially increases the risk of disease spread. Likewise, disease surveillance is restricted to *Brucella abortus* and excludes other *Brucella* species of importance, such as *B. melitensis* and *B. suis*. The countermeasures are mostly focused on cattle and only a few actions are in place for the management of brucellosis in other livestock species. In humans, cases of brucellosis are annually diagnosed, although the disease remains highly underreported. High impact educational and training programs are required to address the disease in a comprehensive manner, including vulnerable groups, such as traditional smallholders and low-productivity regions, as well as other stakeholders, such as healthcare and veterinary authorities. Important financial investments based on sustained cooperation between governmental institutions, industry, and farmers are important for developing affordable and effective strategies to control the disease.

## Introduction

Brucellosis is a zoonotic disease of nearly worldwide distribution, considered to be endemic in the Mediterranean, North and East Africa, the Middle East, South and Central Asia, and Central and South America (1). It is caused by gram-negative bacteria from the genus *Brucella*. The most common species that affect livestock and humans are *B. abortus, B. suis*, and *B. melitensis*, which preferentially (but not exclusively) infect cattle, swine, and small ruminants, respectively (2). Other *Brucella* species include *B. ovis* (sheep), *B. canis* (dogs), *B. neotomae* (rodents), *B. microti* (voles), *B. pinnipedialis* (pinnipeds), *B. ceti* (cetaceans), *B. papionis* (baboons), *B. vulpis* (foxes), and *B. inopinata*, which was isolated from a human breast implant (3).

Abortion and infertility are the most common clinical signs in animals (4). In cattle, brucellosis is associated with abortions during the last trimester of gestation, retained placenta, and weak newborn calves (5). Similar clinical signs occur in small ruminants, although the disease is more severe in goats than in sheep. Mastitis is a common complication in caprine brucellosis. In swine, early fetal loss occurs, and abortion is less common than in cattle. In males, testes and accessory glands are usually affected (6). Contact with infected fetal membranes, aborted fetuses, and uterine secretions allows the dissemination of the pathogen (7). Brucellosis has been recognized as a pathogen in livestock of significant importance. Direct effects include economic losses due to abortion, decreases in milk production, veterinary treatment costs and premature death or culling of infected animals as well as reduced fertility, infection of offspring and transmission to uninfected domestic or wildlife animals. Indirect effects mainly include costs incurred for the implementation of control programs and the forgone revenue due to restricted access to international markets (8).

As a zoonotic disease, brucellosis causes a debilitating illness in humans (9) with significant morbidity in endemic areas (10). The disease is characterized by fever, fatigue, sweats, and malaise. Complications, such as arthritis, endocarditis, and neurological disorders may occur (9). The consumption of raw milk and/or unpasteurized dairy products is the main risk factor for human infections (1). Veterinarians, laboratory workers, livestock keepers, abattoir employees and those associated with animal product industry have a higher risk of acquiring the disease through occupational exposure (4, 5). Effects of human brucellosis involve healthcare costs, loss of productive years, physical pain and emotional suffering, which reduce the quality of life for infected people. Moreover, the disease has a negative impact on the overall human population due to loss of livestock production which is a threat to food security (8).

The aim of this scoping review is to provide an overview of the brucellosis status in Colombia and the factors associated with its persistence, to highlight the strengths and gaps of the adopted countermeasures, with the overall goal of supplying evidence to policy-makers on the most suitable approaches to mitigate the disease burden. It is important to note that most of the available data of brucellosis in Colombia is focused on cattle. In other livestock species, few studies have been conducted and limited actions are taken to manage the disease. Consequently, little is known in this field, which represents a limitation of this scoping review.

## Methods

A literature search was conducted in different databases (Ovid MEDLINE, CAB, Global Health, FSTA, EMBASE, Medic Latina, Fuente Académica Plus, and Agrícola). The most common terms used in the search strategy were Brucel^*^ and Colombia. 148 publications were retrieved and those with available abstracts were evaluated. There were no restrictions on language, type of study design or year of publication and, considering the limited information existing, we used broad inclusion criteria (brucellosis in Colombia and prevalence, epidemiology, livestock species, risk factors, economic impact, public health, among others) in our searches. Articles were mainly rejected when (i) they were focused on other countries or diseases and/or (ii) the study population was different from livestock species or humans. Based on these criteria, 27 articles were selected and summarized for data extraction. Due to the heterogeneity of the studies, different aspects were included in the data extraction (e.g., type of study, location, period of study, population, sample size, livestock production system, diagnostic tests and prevalence, main conclusions and additional relevant data). We compared the annual prevalence based on official data, which includes animals from different livestock species distributed in most of the country. We also considered an independent study that tested cattle from all regions following the official established protocols.

Another relevant source of information was the website of the Colombian Agriculture and Livestock Institute (*Instituto Colombiano Agropecuario*, ICA). Livestock census, regulation of brucellosis, annual reports of the disease among other information were retrieved from this platform. Gray literature was included in our review, due to its importance in identifying and understanding many aspects of the disease in the country. Available data of human brucellosis in Colombia is very limited. Among a few publications found, we included one that collected information from 2000 to 2012 (11). Additionally, a publication about undifferentiated febrile illness was also considered (12).

### Colombia and Livestock Systems

Colombia is a tropical country located in the north of South America. It is bordered by Panama to the northwest, by Venezuela and Brazil to the east and by Ecuador and Peru to the south. The country has a continental land area of 1,141,748 km^2^ and is divided into 32 departments and five geographic regions (Caribbean, Pacific, Andean, Orinoquia, and Amazon) based on topography and weather conditions (13). In Colombia, agricultural activities play a significant role in the socioeconomic development of the country (14). The contribution of the agriculture sector to the GDP (Gross Domestic Product) has increased in recent years, from 5.3% in 2013 to 6.3% in 2017 (15). In 2018, it was the second economic area of highest growth, despite declines in previous decades. Historically, the agricultural sector contributed up to 20% to the GDP, highlighting its importance to the nation in the 1980s and 1990s (16).

**Figure 1:**
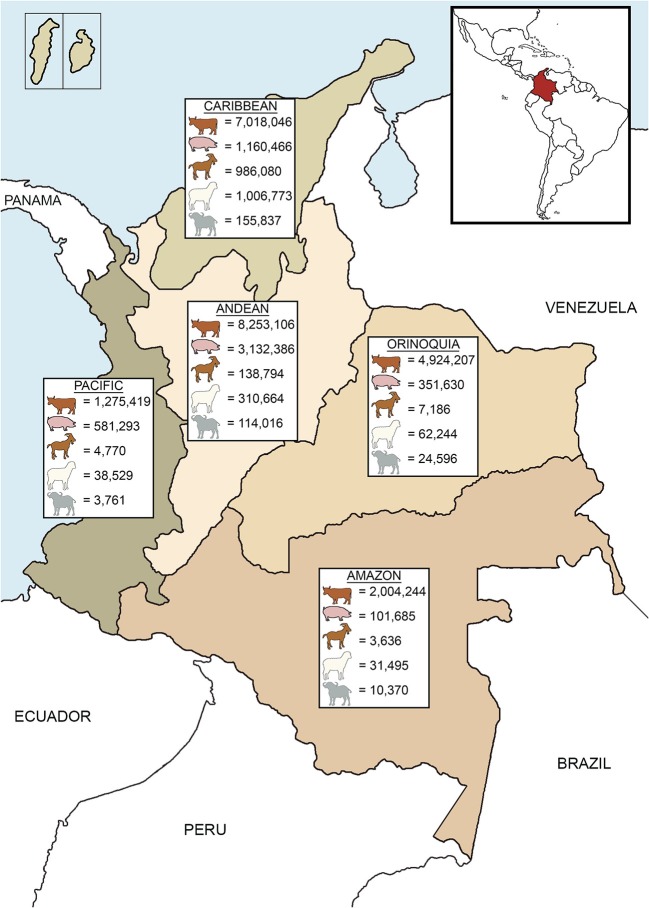
Livestock distribution according to the geographic regions of Colombia. Livestock populations are based on official data of the national census in 2017 (17).

Livestock species in Colombia include cattle, swine, small ruminants (sheep and goats), and buffalo (*Bubalus bubalis*), which are distributed throughout the country (Figure 1). According to the national livestock census of 2017 conducted by the Colombian Agriculture and Livestock Institute (*Instituto Colombiano Agropecuario*, ICA), Colombia contains 23,475,022 bovines, 5,327,460 pigs, 1,140,466 goats, 1,449,705 sheep, and 308,580 buffalo (17). Cattle are the most populous and common livestock species (17) and contribute 21.8% of the total agriculture GDP, and 48.7% to the livestock GDP (18). Dairy cattle systems are mostly located in the Andean region (19) while Caribbean and Orinoquia regions hold the majority of beef cattle (17). Mixed production systems (dairy and beef) are mostly distributed across the northern and southeastern portions of the country (19). Cattle are reared in pastoral farming and it is estimated that 43.7% of the producers have <10 animals, 37.2% between 11 and 50, 15.9% between 51 and 500, 2.9% between 500 and 1,000, and only 0.3% of the farmers have more than 1,000 animals (20). In dairy cattle systems the average milk production per cow ranges between 12 and 14 L/day, although some farms produce up to 27 L/cow daily. Animal density is around 1–2 cows/hectare, and with ~99,000 producers, these systems hold 40% of the milk production of the country (19, 21). Double purpose animals are handled by 250,000 farmers, approximately. Although in mixed systems milk production per cow is lower (around 3–5 L/day), these systems denote 60% of the national production. Milk collection centers process 48% of the total production, 30% is used by industry for the elaboration of by-products, 13% is sold in farms as raw milk and cheese, and 9% is intended for calves and family consumption, mainly in small farms (19). Beef cattle production systems comprise about 50% of the total cattle inventory in Colombia, with an estimated density of 0.6 animals per hectare (20). Around 4.7 million beef cattle belong to feedlot systems, whereas 9.1 million are raised in farms until the last stage (22). Between 2001 and 2010, the bovine population increased by 13.5%. However, adverse climatic conditions negatively affected the productivity and cattle population decreased by 8.3% from 2010 to 2016 (18). More recently, cattle numbers have begun to recover and increased by 3.5% from 2016 to 2017 (18, 23).

In addition to cattle, pigs are an important livestock species and are increasingly popular. Between 2005 and 2017, the population of pigs roughly doubled from 2.5 to 5.3 million (24). Pig production is mainly concentrated in the Andean region (17) (Figure 1); the majority (two-thirds) are reared intensively by commercial farms while the remaining third is held by small-scale farmers, having <10 sows (24). Small ruminants are the mainstay of income for traditional farms in the Caribbean region (17, 25) (Figure 1). Meat is the primary source of income, but other commodities include milk (used for local consumption or cheese elaboration) and wool (sold in artisanal markets). Additionally, trade of live animals can be a source of income to producers (26). Small ruminants are reared in extensive systems, with few exceptions of semi-intensive systems (25). Populations of small ruminants saw a 9.8% decline from 2009 to 2015 (26, 27), but numbers have rebounded (6.2% growth) between 2015 and 2017 (17, 27). Buffalo were introduced to Colombia in 1960 (28) and are mainly located in the Caribbean and Andean regions (Figure 1). Buffalo production has increased 19.3% between 2016 and 2017 (17, 23) and is rapidly becoming an important livestock species due to its adaptability to tropical conditions and the economic benefits of meat and milk production (28, 29).

Mixed extensive livestock systems are common in the country and belong mainly to smallholders, who use part of their production for self-consumption and contributing to their livelihood (30). These family livestock systems play a major role in sustained rural development and account for up to 80% of the farms in the country (31).

### Status and Current Management of Brucellosis

In Colombia, brucellosis was first serologically diagnosed in 1924, and was isolated for the first time in 1927 (32). Outbreaks in herds have been reported for almost a century (33). The only recognized *Brucella* species in livestock is *B. abortus* (biovars 1, 2, and 4) (34). The other only known strain to be present in the country is *B. canis*, which infects dogs (35). Interestingly, despite the increase in population of other livestock species, such as swine and small ruminants, there have been no reports of *B. suis* or *B. melitensis* in Colombian livestock (36). This is probably due to the small number of pigs, sheep and goats that are tested and then only for *B. abortus* (37).

Acknowledging the endemic status of the disease, in 2002, the ICA decided to implement a national program for the control, prevention, and eradication of brucellosis in cattle, buffalo, goats, sheep, swine, and equids (37). The countermeasures listed in the plan include: (1) mandatory immunization of bovine and bubaline females, (2) mandatory notification of suspected and positive animals, (3) epidemiological surveillance, and 4) control of animal movement. The live attenuated vaccines strain 19 (S19) and strain RB51 are the officially approved vaccines in Colombia for the control of bovine brucellosis. Vaccination using one of these strains is mandatory for all bovine and bubaline females aged between 3 and 8 months (37). In 1999, almost 30 years after the beginning of vaccination with S19, the RB51 vaccine was introduced into the country (38) due to its DIVA (differentiating infected from vaccinated animals) capabilities (39). However, this vaccine is more expensive than S19, and its efficacy has been shown to be lower (3, 40–42). Therefore, females immunized with RB51 must be booster-vaccinated with the same strain between 13 and 18 months. In Colombia, this revaccination is optional in animals immunized with S19 (37), an adopted practice that is not stipulated under the World Organisation for Animal Health (OIE) guidelines (43). Booster-vaccination should be done only with RB51 vaccine in non-pregnant females older than 5 years of age and thereafter, every 5 years. Vaccination is carried out during two specific periods per year as established by the ICA. If some animals do not meet the vaccination requirements during these cycles, farmers can request a permit to immunize them outside of these periods which is called “strategic vaccination” and is only done by authorized veterinarians (37). Vaccination with S19 is subsidized by the Colombian Federation of Cattle Producers (FEDEGAN). In contrast, expenses of vaccination with RB51 are completely covered by cattle owners (44) which is around US $1.55 per animal.

As member country of the OIE (45), Colombia follows their list of notifiable terrestrial and aquatic animal diseases (46). Therefore, clinically suspected brucellosis cases are reported to the local veterinary authority (45) which confirms suspected cases by using approved diagnostic tests (37). Unfortunately, due to the absence of specific clinical signs, lack of awareness, and similarity to other endemic diseases that cause reproductive failure, farmers tend to self-medicate resulting in underreporting of brucellosis (47, 48). As a solution to improve diagnostic availability, the so-called “Authorized Inspection Organisms” were created, which are private entities across the country, where specific laboratories and trained veterinarians are added to a list of authorized individuals and entities which are approved to diagnose the disease (13, 49). Unfortunately, the low income of many small-farmers and the relatively high costs of the assays do not allow for the testing of many clinically suspected animals, contributing to the underestimation of the disease.

**Figure 2:**
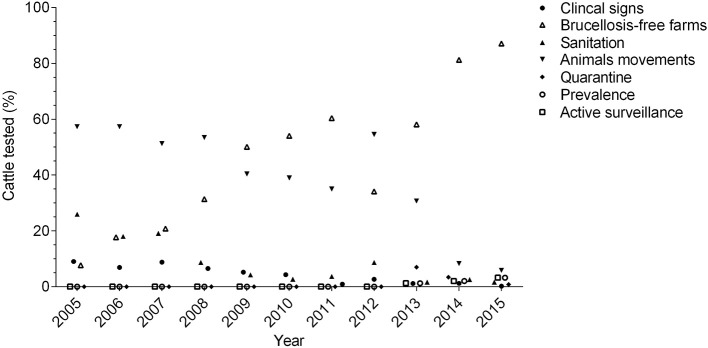
Distribution of cattle tested between 2005 and 2015, according to the testing criteria (testing for analysis of prevalence, quarantine for international trade, and active surveillance is reported since 2013).

On farm detection involves the use of a screening test, followed by a confirmatory test. Testing is mandatory for international trade, animal movements inside the country and for clinically suspected animals (37, 45, 50). Two protocols are officially approved for the diagnosis of *B. abortus* in bovine and bubaline females older than 24 months and bulls older than 8 months. The first approach utilizes the Rose Bengal Plate Test (RBPT) or Indirect ELISA (except in buffalo and bovine immunized with S19 and booster-vaccinated with RB51) as a screening test, followed by Fluorescence Polarized Assay test (FPA) as a first confirmatory and Competitive ELISA as a second confirmatory approach. The second method utilizes FPA as a screening test and Competitive ELISA as a confirmatory test (37). Swine, sheep, and goats older than 6 months are only tested for *B. abortus* with RBPT as a screening test and Competitive ELISA as a confirmatory test (37). There is no official surveillance for *B. melitensis* and *B. suis*, highlighting a significant gap in the disease surveillance. Consequently, the lack of an appropriate epidemiologic surveillance strategy for other *Brucella* species cannot truly confirm the claimed “free status of *B. melitensis* and *B. suis*” for the country. Microbiological culture and molecular detection methods are not used routinely (37) due to the lack of laboratory facilities to perform these assays, posing a significant impediment. Producers must slaughter animals confirmed to be positive without receiving any indemnity. The only exception was in 2012, when US$ 2.0 million were allocated to compensate cattle owners for slaughtering test-positive animals. However, this budget only allowed the slaughtering of 13,249 animals (cattle and buffalo) from more than 70,000 positive reactors during that year (51, 52). Usually, farmers are not compensated (13) and consequently, many producers who are not willing to sacrifice their animals do not test clinically suspected cattle, failing to remove positive animals (48). In fact, the lowest percentage of cattle tested annually corresponds to those with suspected clinical signs (Figure 2) (36, 51, 53–61). These animals may be empirically treated with antibiotics, illegally displaced to other farms, or sacrificed in non-authorized slaughterhouses, perpetuating the disease.

As part of epidemiological surveillance, a program to certify farms free of brucellosis was established, and since 2009, this program covers most of the cattle tested annually (Figure 2) (36, 51, 53, 58–61). Farms are certified when 100% of the animals are found to be negative for *B. abortus* after being tested twice in an interval of 6 months. To maintain this status, a re-certification is done after 1 year and thereafter, every 2 years. For this re-certification, animals are randomly tested, and the sample size depends on the number of animals that meet the sampling age in the farm (e.g., <16: all, 101–150: 66, >1,000: 100), except in dairy farms, where all animals are tested. If a farm has positive animals during the certification or re-certification, positive reactors are sacrificed, the farm is placed under quarantine and animals from neighboring farms are tested, which is known as “sanitation” (37). However, the costs associated with this program (e.g., test and slaughter without compensation) is a limiting factor for many producers. Therefore, participation in the program is voluntary and the monetary incentive consists of an increase in the price of milk sold by the farm (US $0.0032 per liter of milk) if they are declared negative to brucellosis (62). Beef operations do not have any incentive. This explains why most of the participants in the program are dairy cattle and double purpose farms, while participation by beef cattle systems is very limited (13). In fact, in regions were beef cattle are predominant, surveillance activities are lower than in those that hold mainly dairy cattle (63). The number of active farms in the brucellosis free program has declined significantly in recent years, from 20,932 farms in 2015 to only 15,676 farms in 2018 (52). The current estimated number of cattle farms in Colombia is about 514,000 (17) which means that the program has successfully certified <4% of the herds (52). Since 2013, active surveillance is carried out in cattle, although during that year, it represented only 1.2% of the total cattle tested (61). However, these activities have been slowly increasing, and in 2015, 3.4% of the testing in this species corresponded to active surveillance (Figure 2) (36).

Livestock movements within the country are only authorized if animals are serologically negative to brucellosis (64). Brucellosis-free farms can move their animals without restriction. In contrast, farms positive for brucellosis or those with unknown disease status are only able to move animals if they have a negative serological test issued up to 30 days prior to movement (37). During 2005 and 2008, up to 50% of the total cattle tested corresponded to animal movements, and more than 110,000 animals were tested annually during those years (54–57). More recently, these numbers have declined, and in 2015, only 23,133 cattle were tested for this activity, which represented 5.9% of the total cattle tested that year (Figure 2) (36). Legally imported livestock are clinically examined at all entry points. If their entry into the country is authorized, animals are placed in quarantine zones where they undergo laboratory tests to confirm their health status. Only after being found free of infectious diseases, including brucellosis, are animals allowed to be moved to their final destinations (50). It is well-known that non-regulated animal trade has important implications in the dissemination of brucellosis (65). However, despite legislation, illegal animal movement occurs inside the country, although the exact numbers are unknown (63). Illicit importation of animals that do not meet entry requirements is a current concern. In fact, smuggling of cattle from Venezuela has become very cost-effective due to the devaluation of its national currency, which allows the purchase of Venezuelan livestock at low prices for a profitable sale in Colombia (66). Between 2016 and 2018, ~4 million cattle were introduced into the country (67), and according to official data, the number of mobilized cattle exceeds the total number of animals legally registered with the ICA, especially in regions bordering Venezuela (68).

Brucellosis prevalence, as measured by confirmatory serological assays, and the number of animals tested (cattle, sheep, goats, swine, and buffalo) has fluctuated over the years, and a clear trend has not emerged from the data (Table 1). Between 2005 and 2015, the sample size as well as the test and cull programs were affected by climate-related emergencies (13, 36, 51, 53–61). Therefore, the serological data should be interpreted cautiously because these variations in sample size can influence the seropositivity.

**Table 1:** Number of animals tested (cattle, sheep, goats, swine, and buffalo) and percentage of seropositivity between 2005 and 2015.

**Year**	**Animals tested**	**Seropositivity (%)**
2005	199,429	5.2
2006	232,426	4.7
2007	242,013	4.6
2008	307,784	4.3
2009	779,105	2.8
2010	427,873	5.7
2011	561,904	6.1
2012	1,528,324	4.6
2013	763,707	3.2
2014	338,651	4.2
2015	404,243	3.4

**Figure 3:**
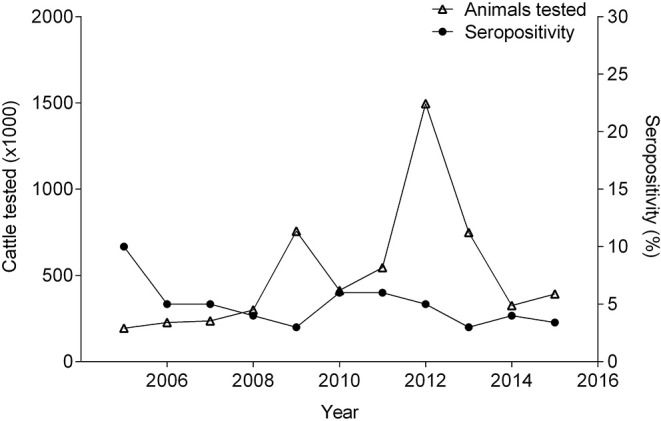
Number of cattle tested and percentage of seropositivity between 2005 and 2015.

Cattle have strongly influenced the livestock seropositivity to brucellosis, due to its high contribution (up to 98%) in the numbers of animals tested annually (36, 51, 53–61). In 2005, 10% of the cattle tested were positive (57). Between 2006 and 2015, the sample prevalence remained between 2.8–6.1% (Figure 3). During those years, annual fluctuations in the herd-level prevalence of brucellosis were recorded (15–28%) (36, 51, 53–61). Since 2013, the ICA has begun to report population prevalence estimates. However, these estimates are derived from disproportionately small sample sizes, comparatively with the population size (36, 60, 61). A recent study, which evaluated 5,215 cattle, found a seroprevalence of 1.5% (69). This study calculated the sample size to be representative for each region; however, as well as the ICA, the study tested both dairy and beef operations without considering differences in herd management like biosafety practices, herd density, and geographical conditions that can affect transmission risks; which is a limitation. Therefore, different epidemiological surveillance approaches are needed to identify risk factors for both dairy and beef operations in order to effectively manage brucellosis in these populations. Furthermore, this study exposed the difficulty of applying the FPA as a brucellosis screening tool because if the cut-off value is not determine carefully, then, the test will not be able to differentiate between animals vaccinated with S19 versus those that had been exposed to a wild-type strain (69). In 2018, the Department of Antioquia (northeast of the country) declared an outbreak of brucellosis (mainly in dairy cattle) (70), and speculated that factors, such as vaccination failures and unrestricted movement of cattle from other regions were responsible. Notwithstanding these contingencies, it is also possible that because the cut-off value was arbitrarily lowered in the confirmatory test, more animals were considered positive. More science-based evidence is needed to validate the accuracy of the new cut-off value. Unfortunately, an epidemiologic study has not been published, and the region remains under quarantine (70).

**Figure 4:**
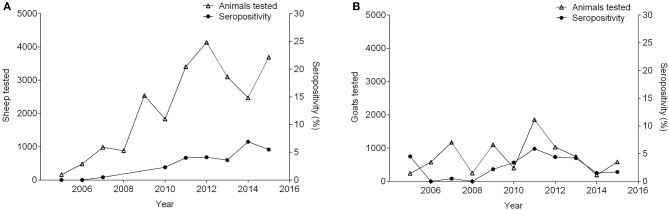
Number of small ruminants tested and percentage of seropositivity between 2005 and 2015. **(A)** Sheep (seropositivity not available in 2008 and 2009), **(B)** Goats.

**Figure 5:**
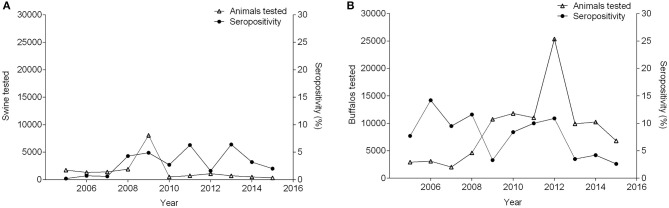
Number of pigs **(A)** and buffalo **(B)** tested and percentage of seropositivity between 2005 and 2015.

Between 2005 and 2015, seropositivity in sheep increased from 0 to 5.5%, while the number of sheep tested increased by 22-fold. During this time period, in the goat population, sample size, and seroprevalence also varied (Figure 4). Concurrently, pigs experienced variation in seropositivity and sample size along with buffalo (Figure 5) (36, 51, 53–61). There was not a correlation between the seropositivity, and the animals tested.

Few small-scale serological studies of brucellosis prevalence have been conducted in Colombian livestock (Table 2). The reported seroprevalences in cattle have ranged between 0.6 and 6.3% at individual level, and 12.7–40% at herd-level (71–80). In buffalo, two different studies assessed the seroprevalence of brucellosis by RBTP and Competitive ELISA, finding a higher seropositivity in buffalo from the Amazon region, than in those reared in the Caribbean region (11.9 and 3%, respectively) (29, 80). It is hazardous to state a conclusion from these studies, due to the heterogenicity in their conditions. Only one study has been conducted to assess the status of *B. melitensis* in small ruminants, which did not identify positive reactors (81). Likewise, the only study conducted in pigs to evaluate the presence of *B. suis*, established 0% of seropositivity (82). This evidence shows that most of the investigations have been focused on cattle, leading to a paucity of research in other livestock species and other *Brucella* species than *B. abortus*.

**Table 2:** Studies of brucellosis seroprevalence in Colombian livestock.

**Region [department(s)]**	**Population of study**	**Diagnostic test(s)**	**Prevalence**	**References**
Amazon (Caquetá)	172 cows and 15 bulls from 20 farms	RBPT and Competitive ELISA	Cows: 5.8% Bulls: 0% Farms: 40%	(71)
Caribbean and Andean (Atlántico and Antioquia)	749,220 cattle from 32,872 farms	RBPT, Indirect ELISA and Competitive ELISA	Cattle: 5.8% Farms: 27.9%	(72)
Andean (Cundinamarca)	546 cows from 46 farms	Competitive ELISA	Cattle: 4.2%	(73)
Caribbean (Córdoba)	29,227 cows and 742 bulls from 4,922 farms	RBPT, Indirect ELISA and Competitive ELISA	Cattle: 3.7% Farms: 12.7%.	(74)
Caribbean (Magdalena and Bolívar)	146 cattle from Bolívar 100 cattle from Magdalena	RBPT and Competitive ELISA	Magdalena: 6% Bolívar: 0.6%	(75)
Caribbean and Andean (Bolívar, César, Norte de Santander and Santander)	174 bulls	Indirect ELISA	4.02%	(76)
Caribbean (Córdoba)	384 cows	RBPT and Indirect ELISA	6.3%	(77)
Caribbean (Córdoba)	1,413 cows	RBPT and CFT	Cattle: 3.4% Farms: 25%	(78)
Andean and Caribbean	4,144 cows	RBPT and SAT	3.3%	(79)
Amazon (Caquetá)	Cattle: 297—Buffalo: 289 from 2 cattle farms, 2 buffalo farms and 3 mixed farms (cattle and buffalo)	RBPT and Competitive ELISA	Buffalo: 11.9% Cattle: 5.3%	(80)
Caribbean (Córdoba)	133 buffalo	RBPT and Competitive ELISA	3%	(29)
Caribbean (Cesar and Sucre)	Cesar: 209 goats from 10 farms Sucre: 120 sheep from 4 farms	RBPT and Indirect ELISA	0%	(81)
Caribbean (Bolívar)	44 pigs	RBPT	0%	(82)

*The studies were summarized according to the region of study, the population tested, the diagnostic tests used and the calculated prevalence (%)*.

*RBPT, Rose Bengal Plate Test; CFT, Complement Fixation Test*.

### Public Health Relevance

Brucellosis is one of the most important zoonoses in Latin America (83), and human cases of the disease have been reported in Colombia over the years (11). Despite its known high prevalence in animals (63), in 2015, only 656 serum samples from suspected human cases were tested. An alarming seropositivity rate of 3.8% was discovered (36). Unfortunately, the sources of infection could not be determined due to gaps in the recorded data (11, 36). Traditionally, the consumption of raw milk and/or its by-products, as well as contact with infected animal tissues and secretions, are considered the main sources for human infection (1). In many regions of the country, artisanal cheese elaboration for local consumption is done without milk pasteurization (19, 26). Non-specific symptoms coupled with a long list of differential diagnoses make human brucellosis a highly underreported disease (2). In fact, with the emergence and re-emergence of febrile illnesses, such as dengue, Chikungunya, yellow fever, Zika virus infection, and Venezuelan equine encephalitis, among others (which share clinical signs with brucellosis), such diseases are usually the main differential diagnoses for febrile illnesses, leaving brucellosis highly underdiagnosed (2, 12). In Colombia, human brucellosis is a clear example of a neglected disease. Despite its public health significance, the establishment of an adequate surveillance system is currently lacking (84). Therefore, it is imperative that a strategy be developed that involves public awareness for consumers and human health workers coupled with enhanced laboratory diagnostic capacity and enhanced surveillance systems for disease recognition.

### Current Challenges

Despite the countermeasures adopted by local authorities 20 years ago, brucellosis is still prevalent (63), and the control of the disease represents a challenge for veterinarians and public health authorities. The difficult diagnosis based on clinical signs (2) and the lack of indemnities for test-and-slaughter (13) make brucellosis an underreported disease in livestock. In humans, the underdiagnosis is attributable to the lack of awareness to consider brucellosis in the list of differential diagnoses of febrile illnesses (85). Current issues associated with the limited epidemiologic studies and surveillance strategies for *B. melitensis* and *B. suis* are also of important concern due to the remergence of these pathogens in South America (81).

There is a direct association between the financial resources of countries and their brucellosis status. The lack of funding allocated for brucellosis programs in developing countries does not allow the execution of strict and effective measures (47). This is important in Colombia, where the decline of the agricultural contribution to the GDP and the growth of other economic activities during the last decades have led to a reduction of public investments into this sector. In addition, the armed conflict that forced the migration of communities from rural areas, the lack of public policies benefiting small and medium-sized farmers, and the establishment of Free Trade Agreements (FTA) not favoring local production, have hindered agricultural activities (14, 86, 87). Poverty in rural areas is a significant barrier to control the disease (85). For instance, when producers are unable to pay for diagnostic testing and slaughtering of positive animals without indemnity, their participation in the program for brucellosis-free farms is not feasible. It is necessary to understand the different socio-economic conditions in the production systems, to design affordable measures and policies for all the producers (20). Budgetary constraints restrain compensations and financing for diagnostic tests, leading to deficiencies in epidemiologic surveillance, and revealing the economic weakness of the program (47). This aspect is relevant due to the fact that effective surveillance must be the strongest component to manage the disease, as it identifies the infected populations, quantifies the disease burden, and directs authorities to the best corrective actions (88). In fact, when extra funding was allocated to the program to compensate cattle owners for slaughtering test-positive animals, surveillance activities improved, and the numbers of animals tested increased from 561,904 in 2011 to 1,528,324 in 2012 (51, 58). Since the beginning of the program, several adjustments have been implemented probably due to budget limitations for the adoption of suitable measures and to errors in the formulation of adequate policies (37, 47, 89–92). In fact, the exclusive surveillance for *B. abortus* even in animals which are mainly affected by other *Brucella* species is an example of these controversial strategies. It seems that the current program has lost confidence and popularity due to the lack of incentives for producers, the slow response of the diagnostic laboratory system, and the uncertainty of properly differentiating between a seropositive herd and a truly infected herd.

According to official data, vaccination coverage has been increasing and, currently, ~98% of bovine and bubaline female populations are immunized (93). Despite this apparent high coverage, brucellosis prevalence has not had a significant decline (63), a situation that may be linked to the persistence of infected animals on farms, vaccination failure and/or the unfamiliarity of the real prevalence due to the fluctuation in the number of animals tested per year and possible errors in the interpretation of diagnostic tests. The illegal movement of animals is also relevant in the maintenance of brucellosis (94) since these animals do not meet the health requirements (including vaccination, which means that the coverage could be overestimated), representing a constant threat of introducing the disease (95). Increased surveillance for these activities is required, as well as the implementation of legal penalties imposed on those who participate in illegal trade of animals into the country (48). In addition, it is necessary to enforce restrictions of animal trade; but more relevant, to educate farmers in good agricultural practices to avoid common risk factors, such as the introduction of new animals with unknown sanitary status to livestock systems or the exchange of bulls among neighboring farms (94).

According to data recorded from 25 small and medium-sized farms located in the Andean region, economic losses in milk production ranged between US $588 and US $772 per cow/year, and one infected cow could represent US $2,412 of lost income annually when other parameters were considered (96). The decrease in milk production due to common events like infertility and abortion motivates dairy cattle farmers to support the control of the disease. The extra revenue of milk sold by brucellosis-free farms is also an incentive for their participation (94). In contrast, the restricted knowledge of beef cattle owners about brucellosis, the lack of economic studies, and the absence of financial incentives in these production systems, limit the awareness of the negative impact of the disease as well as any interest to participate in the program (94). The participation of livestock producers in the development and implementation of strategies has been an essential factor in countries where the disease has been successfully controlled (88). Considering that economic incentives increase the involvement of farmers in the control of brucellosis, an active role of industry is critical at this point (85). The development of strategies by public-private partnerships may generate financial sources for the program, allowing a better coverage in terms of financial incentives for producers. Educational strategies for community empowerment and the development of locally based-organizations facilitate the participation of the farmers in the brucellosis program and in generating funding sources for its sustainability (97, 98).

High impact educational and training programs must be focused on all socio-economic levels and livestock systems, including vulnerable groups, such as traditional smallholders and low-productivity regions, where surveillance activities are limited (13). These public awareness campaigns should be tailored to educate consumers and farmers of the risks associated with the consumption of raw milk or its dairy products, and the advantages of reducing the disease burden on farms (48). An active role of healthcare authorities, including qualified professionals in the control and prevention of zoonoses, will lead to a better diagnosis of brucellosis in humans as well as a better understanding of the disease dynamics in affected populations (85). Human morbidity is significant in areas where brucellosis is endemic (10), and the management of the disease in animals represents the best approach to decrease the rate of infection in people (99). Therefore, the development of an effective program with affordable strategies for all livestock owners will contribute to the welfare of communities (96). To effectively increase the impact of the current program, an epidemiologic assessment of the countermeasures in place is necessary. Other strategies include the implementation of differential surveillance strategies for the different production systems, the identification of high-risk areas, and the identification of truly infected farms, which implies a robust laboratory system. Scientific reviews of the efficiency and interpretation of the serologic tests and their cut-off, as well as the further effect of S19 and RB51 vaccination on the results of these assays are also required. Concerted and participatory efforts are critical to perceive the cost-benefit of the program, including important financial investments and sustained cooperation between governmental institutions, industry, and farmers (47).

In spite of a national brucellosis control program, the prevalence of the disease has lacked a trend to decline over the years, revealing the need to reformulate the strategies currently in place. Although governmental entities play a significant role, an active participation of livestock producers as well as industry is important.

## Author Contributions

LA-G wrote the manuscript and performed the literature review. DG-G participated in writing, editing, and literature review. JZ-V is joint senior author and participated in editing. AA-G is joint senior author and participated in its design, coordination, and editing. All authors helped conceive of the manuscript's message and approved the final manuscript.

### Conflict of Interest

The authors declare that the research was conducted in the absence of any commercial or financial relationships that could be construed as a potential conflict of interest.
